# Virtual Reality-Based Visuospatial Training for Older Adults: System Design and Pilot Evaluation of VR RoomSpace

**DOI:** 10.3390/healthcare14172801

**Published:** 2026-09-01

**Authors:** Agatha Maisie Tjandra, Chien-Hsu Chen

**Affiliations:** 1 Department of Industrial Design, National Cheng Kung University, Tainan City 701, Taiwan; 2 Fakultas Seni dan Desain, Universitas Multimedia Nusantara, Tangerang 15811, Indonesia

**Keywords:** virtual reality, cognitive training, mental rotation, spatial orientation, visuospatial ability, older adults

## Abstract

**Background/Objectives**: Visuospatial ability supports everyday tasks, such as judging distance and navigating, yet declines with age, especially after 70. This study presents VR RoomSpace, a virtual reality system combining immersive exploration with paper-based tasks to engage allocentric and egocentric spatial frameworks. **Methods**: Sixty community-dwelling older adults (aged 65–79; M = 69.8, *SD* = 4.4; MMSE = 29.2, *SD* = 1.4) trained for eight weeks. The experimental group (*n* = 31) received four weeks of pen-and-paper training, then four weeks of VR RoomSpace training; the control group (*n* = 29) received eight weeks of pen-and-paper training. The Mental Rotation Test (MRT; allocentric) and Perspective Taking Spatial Orientation Test (PTSOT; ego-centric) were assessed at baseline and evaluation. **Results**: Both groups improved from baseline on both measures. Analysis of covariance adjusting for baseline showed no significant between-group difference in the full sample for the MRT (adjusted difference 1.30, 95% CI [−0.31, 2.91], *p* = 0.113) or the PTSOT (−9.75°, 95% CI [−22.00, 2.51], *p* = 0.117). An exploratory subgroup analysis favored the experimental group on the MRT among participants aged 70–79 (2.29, 95% CI [0.22, 4.37], *p* = 0.031, d = 0.91), but this did not survive correction (Holm-adjusted *p* = 0.251). No differential PTSOT effect emerged in either subgroup. **Conclusions**: This pilot study shows that VR RoomSpace can be delivered to older adults. While primary outcomes were null, the exploratory analyses suggested a potentially greater allocentric benefit among participants aged 70–79, warranting confirmation in adequately powered studies.

## 1. Introduction

The increasing global population of older adults aged 65 years and older highlights the urgency of maintaining cognitive health to sustain independence and quality of life [1]. One important cognitive ability is visuospatial ability, which commonly declines with age as part of normal aging [2,3]. Visuospatial ability refers to the capacity to encode, integrate, and manipulate visual information to construct spatial representations, interpret visual scenes, and mentally transform two-dimensional input into three-dimensional shapes [4,5]. Declines in visuospatial ability can affect older adults’ everyday functioning, including judging depth and distance, descending stairs, detecting objects and movement, maintaining balance, and navigating unfamiliar environments [6,7,8,9]. Furthermore, symptoms of visuospatial decline can be considered an early indicator of dementia [10,11,12]. Therefore, maintaining visuospatial function is critical for preserving older adults’ autonomy in daily life.

From a neurocognitive perspective, the visuospatial thinking process is divided into two frames of reference, namely, the egocentric and allocentric frameworks, which operate interactively in spatial cognition [13,14]. The egocentric framework encodes spatial locations relative to the observer’s own body position. It is dynamically updated as the observer moves through space and supports navigation through judgments in direction and distance [13,15,16,17]. In this study, the egocentric framework was assessed using the Perspective Taking Spatial Orientation Test (PTSOT) [18,19]. The allocentric framework encodes spatial relationships among objects independently of the observer’s current position [13,15]. This framework is closely related to understanding object-to-object relationships, spatial layouts, and shapes, as well as mentally constructing or transforming three-dimensional objects from two-dimensional visual input [20,21]. In this study, the allocentric framework was assessed using the Mental Rotation Test (MRT) [22,23,24,25].

Among non-pharmacological approaches, cognitive training has shown potential for maintaining visuospatial ability in older adults [3,26,27,28,29]. Within the allocentric framework, training approaches have used hand gestures as thinking aids to reduce cognitive load [30,31]. Other studies have applied three-dimensional visualization in virtual reality (VR) to present box stimuli from different angles in the Mental Rotation Test [32]. Within the egocentric framework, an egocentric reference frame encodes locations relative to the observer’s own body [16]. Immersive VR presents environments from a first-person viewpoint, making it suitable for engaging egocentric spatial processing [33]. At the same time, this perspective allows users to understand spatial relationships among objects within a virtual environment [34,35,36,37].

However, most previous visuospatial training studies have targeted allocentric and egocentric abilities separately. The potential of integrating these two frameworks within a single visuospatial training system remains unclear. This integration is theoretically supported because the two frameworks rely on partially distinct neural substrates but interact during spatial encoding [13,38]. Egocentric representations encode spatial information relative to the observer’s position, whereas allocentric representations encode object-to-object relationships independently of the observer’s position [13]. Movement and changes in viewpoint can contribute to the formation of allocentric representations, suggesting that the two frameworks are functionally interconnected rather than independent [13,39]. Integrating first-person spatial interaction with object-to-object encoding may therefore provide complementary spatial experiences within a single training system. Whether this integrated approach provides greater visuospatial benefit than training that does not deliberately engage both frameworks has not been directly tested.

To address this gap, this study developed VR RoomSpace, a VR-based training system that integrates allocentric and egocentric frameworks into a single VR training system. The study evaluated an eight-week visuospatial training by comparing experimental and control groups. The experimental group received pen-and-paper training in the first four weeks and VR RoomSpace training in the last four weeks, while the control group received pen-and-paper training in all eight weeks. The eight-week protocol was selected as a practical duration that provided repeated training exposure while remaining feasible for community-dwelling older adults, where previous shorter interventions also reported measurable training-related changes [40]. We hypothesized that visuospatial frameworks incorporating VR RoomSpace would produce greater visuospatial performance on evaluation than pen-and-paper training alone. As an exploratory analysis, we further examined whether training outcomes differed between participants aged 65–69 and 70–79 years, given evidence of age-related changes in cognitive decline after age 70 [41,42].

## 2. Materials and Methods

### 2.1. Participants

Sixty-three participants aged 65–79 years voluntarily joined this study and were randomized into experimental and control groups. The randomization was generated and maintained by a research assistant who took no part in recruitment, assessment, or delivery of the intervention. Participant information was anonymized before group assignment. The research assistant who generated the sequence was unaware of the study hypotheses and of the intervention objectives.

All participants were screened for eligibility and underwent baseline test assessment. The cognitive screening used the Mini-Mental State Examination (MMSE) [43], which was administered by a licensed psychiatric social worker, to ensure clinical validity in dementia screening. Color vision screening using the Ishihara 14-plate test (Kanehara Trading Inc., Tokyo, Japan) was administered by the research team.

The analysis only includes participants who completed the study and fulfilled the inclusion criteria, such as: (1) aged ≥ 65 years; (2) MMSE score ≥ 23/30, a cut off value considered to provide standard relaxation to the elderly community [44]; (3) no confirmed diagnosis of cognitive disorders, including dementia or Alzheimer’s disease; and (4) a score of ≥12 out of 14 plates on the Ishihara Color Test. The exclusion criteria were (1) completed fewer than three practice cases at the beginning of either the MRT or PTSOT; (2) attended fewer than three pen-and-paper training sessions; (3) attended fewer than three VR RoomSpace training sessions, for participants assigned to the experimental group; or (4) experienced motion sickness during VR RoomSpace training that prevented further participation.

**Figure 1:**
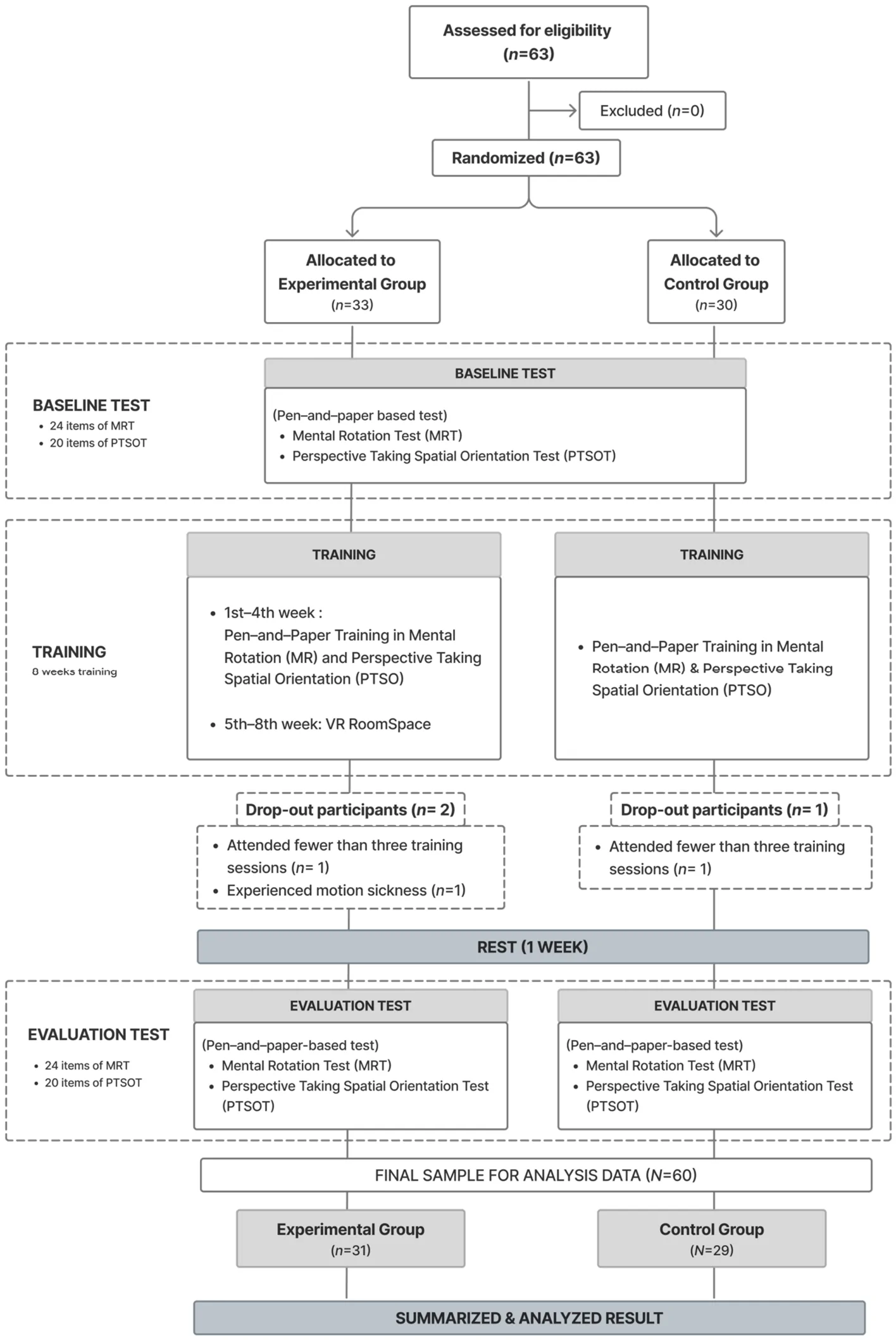
Flow diagram of the study procedure and participant allocation.

During the training, two participants in the experimental group discontinued the experiment; one reported motion sickness during the tutorial session in VR RoomSpace training, and the other attended fewer than three training sessions. In the control group, one participant attended fewer than three training sessions. No data from drop-off participants were analyzed. The dropout rates were 6.1% in the experimental group and 3.3% in the control group, with no significant difference between groups (Fisher’s exact test, *p* = 1.000). Thus, 60 participants remained for the final analysis (experimental group, *n* = 31; control group, *n* = 29) as the full sample. For exploratory analysis, this sample was categorized into age-based subgroups of 65–69 and 70–79 years, according to the prior data analysis of the literature on accelerated decline after age 70 [41,42], as shown in Figure 1.

### 2.2. Environment Setting and Material

**Figure 2:**
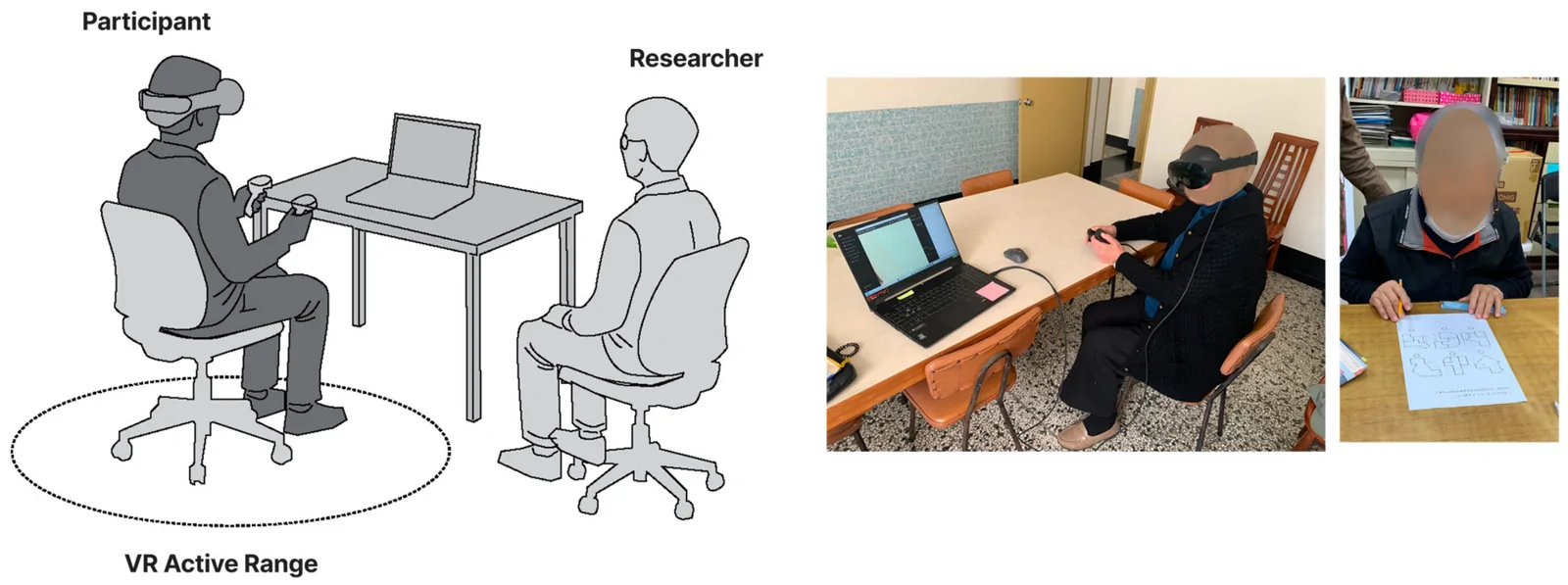
VR RoomSpace experiment set-up.

All VR experiments were conducted in a room in an elders’ activity community center. Each participant was provided with a desk and chair. An Intel core i7 laptop was connected to a Meta Quest Pro (Meta Platforms Technologies, LLC, Menlo Park, CA, USA) head-mounted display, with two hand controllers. The Meta Quest Pro provides a resolution of 1800 × 1920 pixels per eye, a refresh rate of 90 Hz, and a field of view of approximately 106° horizontally and 96° vertically. These display settings were identical for all participants. The place was set up by the research team before the experiment began to adjust each participant’s view. During the experiment, the research team sat next to the participant (out of the VR range area) and guided the training process, as shown in Figure 2.

The MRT was adapted from the redrawn version of the MRT [23,24,25]. The scoring system of each case required identification of two identical figures to the target figure. This test was divided into two parts, consisting of 12 cases with difficulty levels distributed randomly across items. Prior to the test, the participants completed three practice items to ensure task comprehension [45]. The MRT score, representing allocentric ability, was scored as the total number of correct answers, where a higher score indicates better performance.

The PTSOT was adapted from the validated paper version [19] and the computer versions [18,46]. PTSOT performance was quantified as the mean absolute angular error between a participant’s answer and the correct answer, with a lower score indicating better perspective-taking ability. The PTSOT is dissociable from mental rotation and correlates with spatial learning from navigation, which supports its use as a separate measure of egocentric ability [19]. The computerized version reproduces the scoring of the paper version [18]. Published reliability estimates for this test also come from young adult samples.

The MRT and PTSOT were scored using standardized answer keys that produce objective numerical scores. The MRT value is calculated by counting the right answers from the total number of correct answers, with a maximum of 24 points. The PTSOT is calculated as the difference in the angle of error between a participant’s answer and the key answer (angular error). Thus, the lowest degree means the participant’s answer is almost right and means better performance, with a maximum angular error of 180° per case. The total score in the PTSOT counts as the average of the total angular error.

### 2.3. Experimental Procedure

The experiment consisted of three parts: the baseline test, training and the evaluation test (Figure 1). In the baseline test, the participants took the structured pen-and-paper test consisting of the MRT and PTSOT. In the second part, the participants completed training sessions once a week for eight weeks. The experimental group completed four weeks of pen-and-paper training followed by four weeks of VR RoomSpace training, while the control group completed eight weeks of pen-and-paper training. Both groups completed eight training sessions in total. A one-week rest separated the final training session from the evaluation test to reduce fatigue effects carried over from the last session. In the third part, the participants took an evaluation test to check their abilities after training.

### 2.4. Pen-and-Paper Training

The control group received eight weeks of pen-and-paper training, while the experimental group received the same training during weeks one through four. Each session lasted 20 min and included 60 mental rotation items and five perspective-taking items. The mental rotation training items were adapted from the Vandenberg and Kuse Redrawn Test format [23]. During the mental rotation pen-and-paper training, the participants compared two objects and drew a mark to indicate whether they were identical per item. The perspective-taking training items were adapted from the PTSOT [19]. The PTSOT format presented the participants with a series of objects and asked them to imagine standing on one object, facing a second, and drawing an arrow toward a third. Each week, the order to complete the two training types was randomized, so the participants completed either the mental rotation or the perspective-taking items first.

### 2.5. VR RoomSpace Training System

VR RoomSpace was developed using Blender 4.0 (for 3D modeling) and the Unity version 2022.3.18f1 engine and delivered via a Meta Oculus Quest Pro (Meta Platforms Technologies, LLC, Menlo Park, CA, USA) head-mounted display. The participants navigated the virtual environment using a controller. Each VR RoomSpace training session lasted a maximum of 20 min and was conducted once a week.

Each session followed a fixed sequence with a maximum time for every component: a maximum of five minutes for the tutorial, five minutes for exploration of the virtual house, a one-minute break, and three minutes for each of the three paper-based follow-up tasks, totaling 20 min. The participants explored the virtual house once per session. Task difficulty was fixed and did not adapt to participant performance. All participants completed the same components in the same order. A session ended once all components were completed, so time on task varied with individual working pace.

The training began with a tutorial part, in which the participants were briefed on the training objectives and familiarized themselves with moving through the virtual environment using the VR controllers (thumbstick controllers). The participants who reported motion sickness during the VR training were excluded from further participation. During this stage, the research team verbally guided the participants to direct their movement.

The participants then proceeded to the main VR RoomSpace training. This part began with VR RoomSpace exploration in a virtual house. In the exploration phase, the participants viewed the virtual house from a first-person perspective. They perceived the positions of boxes and doors relative to their own viewpoint. This viewpoint-relative coding is the egocentric reference frame [13,47]. Egocentric coding operates while people move through space, not only in static views [38]. As the participants walked through the rooms, they encoded their path and the sequence of views along it.

**Figure 3:**
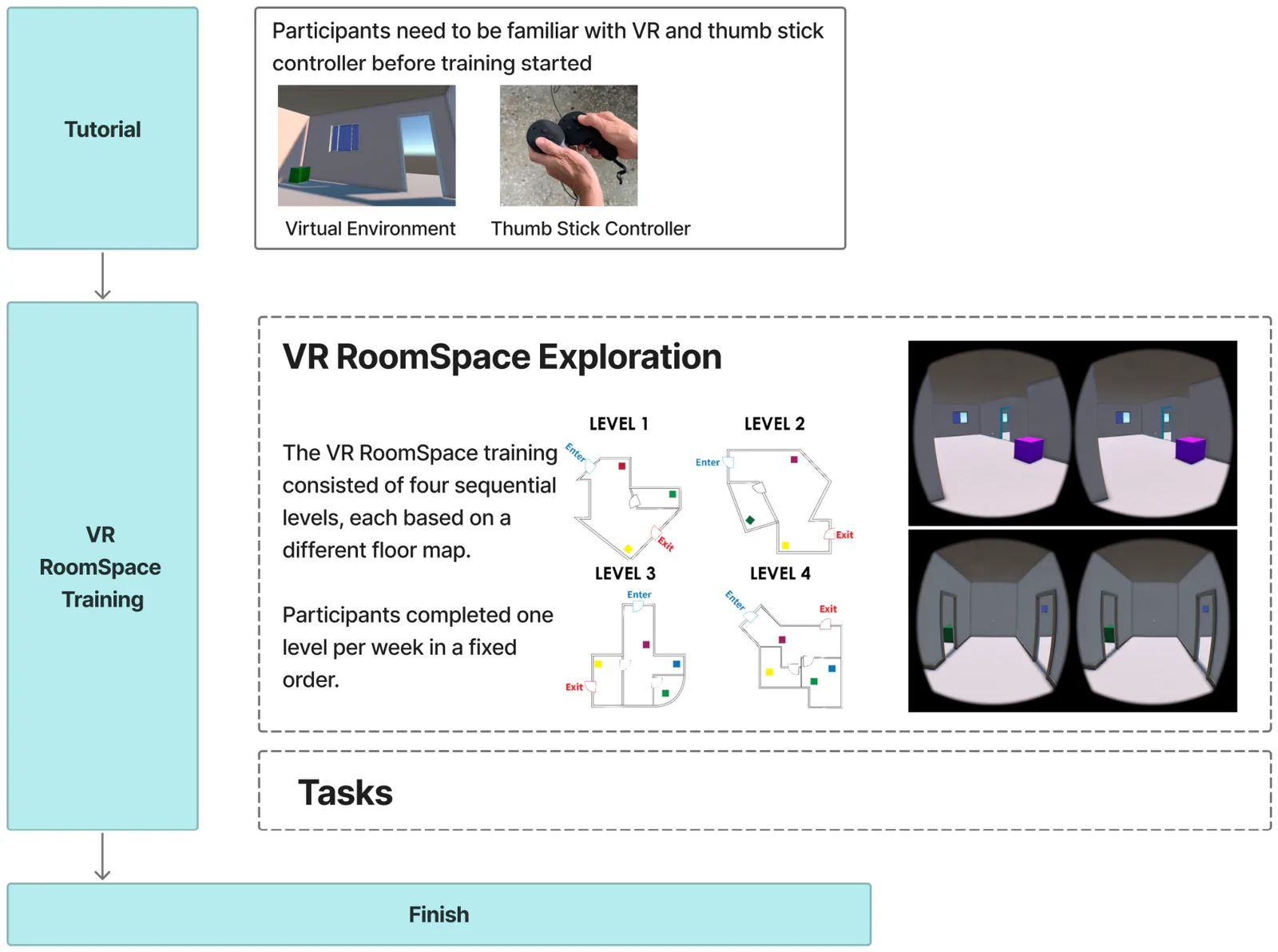
Weekly training procedure of VR RoomSpace.

After the exploration, the participants completed three paper-based follow-up tasks that engaged in allocentric and egocentric frameworks. The first task involved redrawing the floor plan of the virtual house on blank paper, which converts a route-based experience into an allocentric survey representation. Second, the participants chose the most appropriate map of a house from the available options, which required them to compare their memories and first-person experiences with the most similar of the available maps. Third, the participants completed the given floor map with boxes and wrote the color. This required the participants to remember the landmark object-to-object positions. Drawing and reconstruction were chosen in this training because active spatial encoding supports retention more effectively than passive viewing in visuospatial training [48,49]. The VR RoomSpace procedure is shown in Figure 3.

### 2.6. Statistical Analyses

Data analysis was conducted to assess baseline characteristics and evaluate differences in training outcomes between the experimental and control groups. Analyses were performed on the full sample, followed by exploratory analyses of the 65–69 and 70–79 age subgroups and comparisons between these two age subgroups.

All analyses were performed in IBM SPSS Statistics 27, with the significance level set at *p* < 0.05. Descriptive statistics are reported as means with standard deviations. Normality was assessed with the Shapiro–Wilk test for each variable within each group. Mann–Whitney U tests were used for between-group comparisons because normality was violated in the subjects’ age, MMSE, and Ishihara score data.

The primary analysis of the full sample (*n* = 60) used ANCOVA to compare evaluation scores between the experimental and control groups, with the corresponding baseline score as a covariate. The same ANCOVA approach was used for the exploratory age subgroup comparisons and the cross-age comparison within the experimental group. Adjusted between-group differences, 95% confidence intervals (CIs), and standardized effect sizes (Cohen’s *d*) were reported. To account for multiple testing, *p*-values were adjusted using the Holm–Bonferroni procedure (*p*Holm) across the eight between-group comparisons. Effect sizes of 0.20, 0.50, and 0.80 were interpreted as small, medium, and large, respectively. HC3 robust standard errors were used when heteroscedasticity was detected.

The sample size was determined by the number of eligible volunteers available at the time of recruitment. No a priori sample size or power calculation was performed before the study. A sensitivity analysis was conducted after the sample was obtained to characterize the statistical sensitivity of the achieved sample. With 31 participants in the experimental group and 29 in the control group (α = 0.05, two-tailed), the analysis indicated 80% power to detect between-group effects of d ≥ 0.74 in the full sample. The corresponding detectable effects were d ≥ 1.03 for the 65–69 subgroup and d ≥ 1.11 for the 70–79 subgroup.

## 3. Results

### 3.1. Characteristics of Subjects

Sixty participants from the experimental group (*n* = 31) and the control group (*n* = 29) were included in the final analysis. Age-based subgroups (65–69 and 70–79 years) were defined prior to data analysis based on the literature on accelerated decline after age 70 [41,42]. The experimental and control groups did not differ significantly in age, MMSE score, or Ishihara score. This applied to the full sample (Table 1).

The same pattern was observed within each age subgroup. In the 65–69 age subgroup, the experimental group (*n* = 15) and control group (*n* = 17) showed no significant differences (Table 2), and in the 70–79 age subgroup, the experimental group (*n* = 16) and control group (*n* = 12) showed no significant differences (Table 3).

### 3.2. Visuospatial Training Effect in Full Sample

Across the full sample (*n* = 60), both groups showed better performance in evaluation scores on the MRT and PTSOT (Table 4). After ANCOVA adjustment for baseline scores, the experimental group performed better than the control group on both outcomes, but neither adjusted difference was statistically significant: MRT, adjusted difference = 1.30 points, 95% CI [−0.31, 2.91], *p* = 0.113, d = 0.42; PTSOT, adjusted difference = −9.75°, 95% CI [−22.00, 2.51], *p* = 0.117, d = −0.41. The confidence intervals for both adjusted differences are quite wide and include zero, so there is considerable uncertainty regarding the true magnitude of the effect. Because the sample size used was only sensitive enough to detect large effects, the results of this study cannot yet provide definitive conclusions. Therefore, the non-significant results should not be interpreted as evidence that VR RoomSpace has no effect.

### 3.3. Exploratory Analysis Between Age Subgroup Training

#### 3.3.1. Participant Data of the Experimental and Control Groups in the 65–69 Age Subgroup

In the 65–69 age subgroup, both groups showed changes from baseline to evaluation on both measures (Table 5). After ANCOVA adjustment for baseline scores, comparisons showed no significant between-group differences for either the MRT (adjusted difference = −0.23 points, 95% CI [−2.84, 2.39], *p* = 0.861, d = −0.07) or the PTSOT (adjusted difference = −9.34°, 95% CI [−24.57, 5.89], *p* = 0.268, d = −0.36). Although the experimental group had a numerically lower adjusted PTSOT error, the confidence interval included zero. These exploratory findings do not indicate a clear difference between the groups in this age subgroup.

#### 3.3.2. Participant Data of the Experimental and Control Groups in the 70–79 Age Subgroup

In the 70–79 age subgroup, both groups showed better performance in evaluation scores on the MRT and PTSOT (Table 6). After adjustment for baseline scores, the experimental group showed a higher MRT score than the control group (adjusted difference = 2.29 points, 95% CI [−0.22, 4.37], *p* = 0.031, d = 0.91). However, this difference was not statistically significant after Holm–Bonferroni adjustment (*p*Holm = 0.251). For the PTSOT, the adjusted between-group difference was not statistically significant (adjusted difference = −8.55°, 95% CI [−25.88, 8.78], *p* = 0.319, d = −0.39).

These exploratory findings suggest a potentially meaningful advantage of the experimental group in MRT performance, but the evidence was insufficient to establish a statistically significant between-group difference after adjustment for multiple comparisons.

#### 3.3.3. Cross-Age-Subgroup Comparison in the Experimental Group

Within the experimental group, the 70–79 subgroup had lower baseline MRT scores and higher baseline PTSOT error than the 65–69 subgroup (*p* = 0.020 and *p* = 0.003, respectively) (Table 7). After adjustment for these baseline differences, neither the MRT (adjusted difference = 1.15 points, 95% CI [−1.48, 3.78], *p* = 0.377, d = 0.36) nor the PTSOT (adjusted difference = −3.29°, 95% CI [−16.04, 9.46], *p* = 0.601, d = −0.22) showed a significant difference between the age subgroups. These exploratory findings do not provide evidence of a baseline-adjusted difference in training outcomes between the two age subgroups.

In the full sample, baseline-adjusted ANCOVA showed no statistically significant between-group differences for either the MRT or the PTSOT. The confidence intervals were wide and included zero, indicating uncertainty in the magnitude and direction of the between-group effects.

In the exploratory age subgroup analyses, the largest adjusted between-group difference was observed for the MRT in the 70–79 subgroup, favoring the experimental group (adjusted difference = 2.29 points, 95% CI [−0.22, 4.37], *p* = 0.031, d = 0.91). However, this difference was not statistically significant after Holm–Bonferroni correction (*p*Holm = 0.251). No significant baseline-adjusted between-group differences were observed in the 65–69 subgroup or for the PTSOT in either age subgroup. The cross-age comparison within the experimental group also showed no significant baseline-adjusted differences between the two age subgroups.

Overall, the exploratory analyses suggest a possible advantage in MRT performance among participants aged 70–79 years, but the evidence was insufficient to establish a statistically significant between-group difference after adjustment for multiple comparisons. Larger studies are needed to determine whether this pattern is reproducible.

## 4. Discussion

### 4.1. VR RoomSpace Training Effect

This study examined whether VR RoomSpace, which integrates allocentric and egocentric spatial frameworks, provides an additional benefit for visuospatial training in older adults. In the full sample, ANCOVA did not show statistically significant between-group differences in either mental rotation or spatial orientation. The confidence intervals were wide and included zero, indicating uncertainty in the magnitude and direction of the effects. Thus, the findings do not establish an additional benefit of VR RoomSpace over the comparison training, but they also do not establish the absence of an effect. Nevertheless, the more favorable evaluation scores observed in both groups are consistent with evidence that visuospatial ability is malleable and can change with training and experience [50].

In the exploratory analysis, the 70–79 age subgroup revealed a potentially different pattern. This subgroup showed the largest adjusted between-group difference in the MRT, favoring the experimental group (adjusted difference = 2.29 points, 95% CI [0.22, 4.37], d = 0.91). However, this finding did not remain statistically significant after Holm–Bonferroni correction (*p*Holm = 0.251). Therefore, the observed MRT advantage should be interpreted as an exploratory signal rather than evidence of a confirmed age-specific training effect.

In contrast, no statistically significant baseline-adjusted between-group differences were observed in the 65–69 subgroup or for the PTSOT in either age subgroup. These non-significant findings should not be interpreted as evidence of no benefit, particularly given the limited sensitivity of the subgroup samples to smaller effects.

The potential contribution of VR RoomSpace may also relate to its integration of spatial interaction with subsequent paper-based follow-up tasks, including floor plan drawing, map selection, and object placement. These activities required participants to actively encode and reconstruct spatial information, potentially reinforcing spatial representations beyond the VR experience itself. Such active spatial processing may provide a plausible mechanism through which VR-based spatial experiences could support visuospatial learning, although the present study did not directly test this mechanism.

### 4.2. Egocentric and Allocentric Frameworks in VR RoomSpace

VR RoomSpace was designed to integrate allocentric and egocentric spatial processing within a single training program. The results showed different patterns across the two visuospatial outcomes. A baseline-adjusted between-group difference was observed for the MRT in the exploratory 70–79 subgroup analysis, whereas no statistically significant baseline-adjusted between-group difference was observed for the PTSOT in the full sample or either age subgroup. Although the MRT pattern did not remain statistically significant after Holm–Bonferroni correction, the findings suggest that the two visuospatial outcomes may respond differently to VR RoomSpace training. However, this pattern does not establish that one spatial framework is more responsive than the other.

One possible explanation for the different response patterns is that the MRT and PTSOT involve different cognitive demands. The PTSOT is more cognitively demanding than the MRT and recruits working memory and executive control [19,46]. Although VR RoomSpace supports egocentric processing through its first-person perspective [51], perspective taking requires mental reorientation from a specified viewpoint, whereas mental rotation involves mentally transforming an object’s orientation [19,46]. This suggests that perspective taking may require a different training approach and extended exposure or distributed practice to produce measurable transfer to standardized tests. Future studies should examine these possibilities to expand VR RoomSpace training.

## 5. Conclusions and Limitations

This study examined whether VR RoomSpace, which integrates allocentric and egocentric spatial frameworks with paper-based tasks, provides an additional visuospatial training benefit beyond pen-and-paper training alone in older adults. In the full sample, the baseline-adjusted estimates favored VR RoomSpace for both the MRT and PTSOT, but the wide confidence intervals and limited sensitivity of the achieved sample to smaller effects made the findings inconclusive. In the exploratory age subgroup analysis, the 70–79 subgroup showed the largest adjusted between-group difference in the MRT, favoring the experimental group (adjusted difference = 2.29 points, 95% CI [−0.22, 4.37], d = 0.91). However, this difference did not remain statistically significant after Holm–Bonferroni correction (*p*Holm = 0.251). The observed MRT difference should therefore be interpreted as an exploratory signal rather than evidence of a confirmed age-specific training effect. No significant baseline-adjusted between-group differences were observed in the 65–69 subgroup or for the PTSOT. Overall, the study did not establish an additional benefit of VR RoomSpace over pen-and-paper training, but the findings were inconclusive and warranted further evaluation in larger, adequately powered studies.

This study has several limitations. First, the relatively small sample limited statistical power, particularly in the exploratory age subgroup analyses. The full-sample design had 80% power to detect between-group effects of d ≥ 0.74, whereas the observed effects were smaller (d = 0.42 for the MRT; d = −0.41 for the PTSOT). Thus, the non-significant primary results may reflect limited power rather than an absence of a training effect. Second, the study did not assess the long-term durability of training-related changes. Follow-up assessments at one, three, and six months would help determine whether these changes are sustained and whether additional training is needed to maintain them. Third, the findings may have limited generalizability beyond healthy older adults with good physical condition. Participants who experienced motion sickness were excluded, so the results may not extend to older adults with poorer physical conditions or lower tolerance for VR. Finally, the absence of a VR-only control group limits our ability to isolate the effects of cognitive training from those of VR exposure or novelty, and alternative or counterbalanced test materials are required to disentangle these influences.

## Figures and Tables

**Table 1:** Demographic and inclusion assessment data for the experimental and control groups, with no significant between-group differences across the full sample (*n* = 60). Data are reported as mean (standard deviation, *SD*).

Full Data Sample (*n* = 60)	Experimental (*n* = 31)	Control (*n* = 29)	Statistics
Age	70.13 (4.52)	69.55 (4.29)	*U* = 470.50, *z* = 0.31, *p* = 0.759, *r* = 0.04
MMSE	29.19 (1.38)	29.24 (1.46)	*U* = 421.50, *z* = −0.49, *p* = 0.632, *r* = 0.06
Ishihara Color Test	13.87 (0.50)	13.90 (0.41)	*U* = 450.50, *z* = 0.03, *p* = 0.986, *r* = 0.00

**Table 2:** Demographic and inclusion assessment data for the experimental and control groups, with no significant between-group difference in the 65–69 subgroup (*n* = 32). Data are reported as mean (standard deviation, *SD*).

65–69 Age Subgroup Data (*n* = 32)	Experimental (*n* = 15)	Control (*n* =17)	Statistics
Age	65.87 (1.06)	66.35 (1.41)	*U* = 104.50, *z* = −0.93, *p* = 0.363, *r* = 0.16
MMSE	29.20 (0.94)	29.59 (1.00)	*U* = 87.50, *z* = −1.79, *p* = 0.077, *r* = 0.32
Ishihara Color Test	14.00 (0.00)	13.88 (0.48)	*U* = 135.00, *z* = 0.94, *p* = 0.381, *r* = 0.17

**Table 3:** Demographic and inclusion assessment data for the experimental and control groups, with no significant between-group differences in the 70–79 subgroup (*n* = 28). Data are reported as mean (standard deviation, *SD*).

70–79 Age Subgroup Data (*n* = 28)	Experimental (*n* = 16)	Control (*n* = 12)	Statistics
Age	74.13 (2.15)	74.08 (2.39)	*U* = 94.00, *z* = −0.09, *p* = 0.944, *r* = 0.02
MMSE	29.19 (1.72)	28.75 (1.86)	*U* = 117.00, *z* = 1.14, *p* = 0.265, *r* = 0.22
Ishihara Color Test	13.75 (0.68)	13.92 (0.29)	*U* = 91.00, *z* = −0.43, *p* = 0.697, *r* = 0.08

**Table 4:** ANCOVA of the MRT and PTSOT between the experimental (*n* = 31) and control (*n* = 29) groups in the full sample (*n* = 60).

	Score, Mean (SD)			
	Experimental (*n* = 31)	Control (*n* = 29)	Difference Between Means (95% CI)	*p*-Value
**MRT** (**points, maximum 24; higher score = better performance**)				
Baseline	7.26 (2.70)	7.31 (2.98)	−0.05 (−1.52 to 1.41)	0.943
Evaluation	11.19 (3.82)	9.93 (3.27)	1.26 (−0.58 to 3.11)	0.176
ANCOVA	Adj. M = 11.21	Adj. M = 9.91	1.30 (−0.31 to 2.91) d = 0.42	0.113 *p*Holm = 0.788
**PTSOT** (**degrees of angular error; lower score = better performance**)				
Baseline	66.67 (28.89)	73.91 (28.04)	−7.24 (−21.97 to 7.49)	0.330
Evaluation	40.37 (22.95)	55.32 (37.79)	−14.95 (−30.99 to 1.09)	0.067
ANCOVA	Adj. M = 42.88	Adj. M = 52.63	−9.75 (−22.00 to 2.51) d = −0.41	0.117 *p*Holm = 0.788

**Table 5:** ANCOVA exploratory analysis of the MRT and PTSOT between the experimental (*n* = 15) and control (*n* = 17) groups in the 65–69 age subgroup (*n* = 32).

	Score, Mean (SD)			
	Experimental (*n* = 15)	Control (*n* = 17)	Difference Between Means (95% CI)	*p*-Value
**MRT** (**points, maximum 24; higher score = better performance**)				
Baseline	8.40 (2.44)	6.88 (2.47)	1.52 (−0.26 to 3.30)	0.092
Evaluation	11.60 (4.75)	10.35 (3.52)	1.25 (−1.75 to 4.24)	0.402
ANCOVA	Adj. M = 10.82	Adj. M = 11.04	−0.23 (−2.84 to 2.39) d = −0.07	0.861 *p*Holm = 1.000
**PTSOT** (**degrees of angular error; lower score = better performance**)				
Baseline	51.47 (24.35)	76.56 (24.84)	−25.09 (−42.90 to −7.28)	0.007
Evaluation	32.20 (21.85)	60.09 (37.43)	−27.89 (−50.42 to −5.36)	0.017
ANCOVA	Adj. M = 42.05	Adj. M = 51.39	−9.34 (−25.87 to 7.19) d = −0.36	0.268 *p*Holm = 1.000

**Table 6:** ANCOVA exploratory analysis of the MRT and PTSOT between the experimental (*n* = 16) and control (*n* = 12) groups in the 70–79 age subgroup (*n* = 28).

	Score, Mean (SD)			
	Experimental (*n* = 16)	Control (*n* = 12)	Difference Between Means (95% CI)	*p*-Value
**MRT** (**points, maximum 24; higher score = better performance**)				
Baseline	6.19 (2.54)	7.92 (3.60)	−1.73 (−4.11 to 0.65)	0.148
Evaluation	10.81 (2.81)	9.33 (2.93)	1.48 (−0.77 to 3.73)	0.188
ANCOVA	Adj. M = 11.16	Adj. M = 8.87	2.29 (0.22 to 4.37) d = 0.91	0.031 *p*Holm = 0.251
**PTSOT** (**degrees of angular error; lower score = better performance**)				
Baseline	80.92 (25.85)	70.15 (32.82)	10.77 (−11.99 to 33.54)	0.340
Evaluation	48.02 (21.88)	48.56 (38.90)	−0.53 (−24.30 to 23.23)	0.964
ANCOVA	Adj. M = 44.59	Adj. M = 53.14	−8.55 (−25.88 to 8.78) d = −0.39	0.319 *p*Holm = 1.000

**Table 7:** ANCOVA exploratory analysis of the comparison between the 65–69 (*n* = 15) and 70–79 (*n* = 16) age subgroups in the experimental group.

	Score, Mean (SD)			
	Experimental Group			
	65–69 Years (*n* = 15)	70–79 Years (*n* = 16)	Difference Between Means (95% CI)	*p*-Value
**MRT** (**points, maximum 24; higher score = better performance**)				
Baseline	8.40 (2.44)	6.19 (2.54)	−2.21 (−4.04 to −0.38)	0.020
Evaluation	11.60 (4.75)	10.81 (2.81)	−0.79 (−3.63 to 2.06)	0.576
ANCOVA	Adj. M = 10.60	Adj. M = 11.75	1.15 (−1.48 to 3.78) d = 0.36	0.377 *p*Holm = 1.000
**PTSOT** (**degrees of angular error; lower score = better performance**)				
Baseline	51.47 (24.35)	80.92 (25.85)	29.46 (10.98 to 47.94)	0.003
Evaluation	32.20 (21.85)	48.02 (21.88)	15.82 (−0.25 to 31.90)	0.053 U test: *p* = 0.042, r = 0.37
ANCOVA	Adj. M = 42.07	Adj. M = 38.78	−3.29 (−16.04 to 9.46) d = −0.22	0.601 *p*Holm = 1.000

## Data Availability

The data of this study are available from the corresponding author on reasonable request.

## Abbreviations

The following abbreviations are used in this manuscript:

MRT	Mental Rotation Test
PTSOT	Perspective Taking Spatial Orientation Test
VR	Virtual Reality
MMSE	Mini-Mental State Examination

## References

[1] Gianfredi V., Nucci D., Pennisi F., Maggi S., Veronese N., Soysal P. (2025). Aging, Longevity, and Healthy Aging: The Public Health Approach. Aging Clin. Exp. Res..

[2] Bao R., Chang S., Liu R., Wang Y., Guan Y. (2025). Research Status of Visuospatial Dysfunction and Spatial Navigation. Front. Aging Neurosci..

[3] Wahlin T.-B.R., Bäckman L., Wahlin Å., Winblad B. (1993). Visuospatial Functioning and Spatial Orientation in a Community-Based Sample of Healthy Very Old Persons. Arch. Gerontol. Geriatr..

[4] Harada C.N., Natelson Love M.C., Triebel K.L. (2013). Normal Cognitive Aging. Clin. Geriatr. Med..

[5] Waller D., Nadel L. (2013). Handbook of Spatial Cognition.

[6] Fricke M., Morawietz C., Wunderlich A., Muehlbauer T., Jansen C.-P., Gramann K., Wollesen B. (2022). Successful Wayfinding in Age: A Scoping Review on Spatial Navigation Training in Healthy Older Adults. Front. Psychol..

[7] Kalantari S., Mostafavi A., Xu T.B., Lee A.S., Yang Q. (2024). Comparing Spatial Navigation in a Virtual Environment vs. an Identical Real Environment across the Adult Lifespan. Comput. Hum. Behav..

[8] Mak T.C.T., Wong T.W.L., Ng S.S.M. (2021). Visual-Related Training to Improve Balance and Walking Ability in Older Adults: A Systematic Review. Exp. Gerontol..

[9] Muiños M., Palmero F., Ballesteros S. (2016). Peripheral Vision, Perceptual Asymmetries and Visuospatial Attention in Young, Young-Old and Oldest-Old Adults. Exp. Gerontol..

[10] Külzow N., Kerti L., Witte V.A., Kopp U., Breitenstein C., Flöel A. (2014). An Object Location Memory Paradigm for Older Adults with and without Mild Cognitive Impairment. J. Neurosci. Methods.

[11] Puthusseryppady V., Emrich-Mills L., Lowry E., Patel M., Hornberger M. (2020). Spatial Disorientation in Alzheimer’s Disease: The Missing Path From Virtual Reality to Real World. Front. Aging Neurosci..

[12] Quental N.B.M., Brucki S.M.D., Bueno O.F.A. (2013). Visuospatial Function in Early Alzheimer’s Disease—The Use of the Visual Object and Space Perception (VOSP) Battery. PLoS ONE.

[13] Burgess N. (2006). Spatial Memory: How Egocentric and Allocentric Combine. Trends Cogn. Sci..

[14] Possin K.L. (2010). Visual Spatial Cognition in Neurodegenerative Disease. Neurocase.

[15] Berthoz A. (2000). The Brain’s Sense of Movement.

[16] Colombo D., Serino S., Tuena C., Pedroli E., Dakanalis A., Cipresso P., Riva G. (2017). Egocentric and Allocentric Spatial Reference Frames in Aging: A Systematic Review. Neurosci. Biobehav. Rev..

[17] Committeri G., Galati G., Paradis A.-L., Pizzamiglio L., Berthoz A., LeBihan D. (2004). Reference Frames for Spatial Cognition: Different Brain Areas Are Involved in Viewer-, Object-, and Landmark-Centered Judgments About Object Location. J. Cogn. Neurosci..

[18] Friedman A., Kohler B., Gunalp P., Boone A.P., Hegarty M. (2020). A Computerized Spatial Orientation Test. Behav. Res. Methods.

[19] Hegarty M., Waller D. (2004). A Dissociation between Mental Rotation and Perspective-Taking Spatial Abilities. Intelligence.

[20] De Bruin N., Bryant D.C., MacLean J.N., Gonzalez C.L.R. (2016). Assessing Visuospatial Abilities in Healthy Aging: A Novel Visuomotor Task. Front. Aging Neurosci..

[21] Fernandez-Baizan C., Nuñez P., Arias J.L., Mendez M. (2020). Egocentric and Allocentric Spatial Memory in Typically Developed Children: Is Spatial Memory Associated with Visuospatial Skills, Behavior, and Cortisol?. Brain Behav..

[22] Linn M.C., Petersen A.C. (1985). Emergence and Characterization of Sex Differences in Spatial Ability: A Meta-Analysis. Child Dev..

[23] Peters M., Laeng B., Latham K., Jackson M., Raghad Z., Richardson C. (1995). A Redrawn Vandenberg and Kuse Mental Rotations Test—Different Versions and Factors That Affect Performance. Brain Cogn..

[24] Shepard R.N., Metzler J.C. (1971). Mental Rotation of Three-Dimensional Objects. Science.

[25] Vandenberg S.G., Kuse A.R. (1978). Mental Rotations, a Group Test of Three-Dimensional Spatial Visualization. Percept. Mot. Ski..

[26] Bruno J.L., Shaw J.S., Hosseini S.M.H. (2024). Toward Personalized Cognitive Training in Older Adults: A Pilot Investigation of the Effects of Baseline Performance and Age on Cognitive Training Outcomes. J. Alzheimer’s Dis..

[27] Korečko Š., Hudak M., Sobota B., Marko M., Cimrova B., Farkas I., Rosipal R. (2018). Assessment and Training of Visuospatial Cognitive Functions in Virtual Reality: Proposal and Perspective. Proceedings of the 2018 9th IEEE International Conference on Cognitive Infocommunications (CogInfoCom), Budapest, Hungary, 22–24 August 2018.

[28] Korečko Š., Sobota B., Hudák M., Farkaš I., Cimrová B., Vasi’ P., Trojčák D., Hassanien A.E., Shaalan K., Tolba M.F. (2020). Experimental Procedure for Evaluation of Visuospatial Cognitive Functions Training in Virtual Reality. Proceedings of the International Conference on Advanced Intelligent Systems and Informatics 2019.

[29] Silva T.B.L.D., Bratkauskas J.S., Barbosa M.E.D.C., Silva G.A.D., Zumkeller M.G., Moraes L.C.D., Lessa P.P., Cardoso N.P., Ordonez T.N., Brucki S.M.D. (2022). Long-Term Studies in Cognitive Training for Older Adults: A Systematic Review. Dement. Neuropsychol..

[30] Hoe Z.-Y., Lee I.-J., Chen C.-H., Chang K.-P. (2017). Using an Augmented Reality-Based Training System to Promote Spatial Visualization Ability for the Elderly. Univers. Access Inf. Soc..

[31] Lee I.-J., Chen C.-H., Chang K.-P. (2016). Augmented Reality Technology Combined with Three-Dimensional Holography to Train the Mental Rotation Ability of Older Adults. Comput. Hum. Behav..

[32] Lochhead I., Hedley N., Çöltekin A., Fisher B. (2022). The Immersive Mental Rotations Test: Evaluating Spatial Ability in Virtual Reality. Front. Virtual Real..

[33] Tuena C., Mancuso V., Stramba-Badiale C., Pedroli E., Stramba-Badiale M., Riva G., Repetto C. (2021). Egocentric and Allocentric Spatial Memory in Mild Cognitive Impairment with Real-World and Virtual Navigation Tasks: A Systematic Review. J. Alzheimer’s Dis..

[34] Park J.-H. (2022). Effects of Spatial Cognitive Training Using Virtual Reality on Hippocampal Functions and Prefrontal Cortex Activity in Older Adults with Mild Cognitive Impairment. Int. J. Gerontol..

[35] Preston A.M., Padala P.R. (2022). Virtual Reality on the Verge of Becoming a Reality for Geriatric Research. Int. Psychogeriatr..

[36] Skurla M.D., Rahman A.T., Salcone S., Mathias L., Shah B., Forester B.P., Vahia I.V. (2022). Virtual Reality and Mental Health in Older Adults: A Systematic Review. Int. Psychogeriatr..

[37] Wilding R., Barbosa Neves B., Waycott J., Miller E., Porter T., Johnston J., James W., Brajanovski S., Wilson J., Baker S. (2024). Introducing Virtual Reality to Older Adults: A Qualitative Analysis of a Co-Design Innovation with Care Staff. Arch. Gerontol. Geriatr..

[38] Moraresku S., Vlcek K. (2020). The Use of Egocentric and Allocentric Reference Frames in Static and Dynamic Conditions in Humans. Physiol. Res..

[39] Zhou Y., Liu Y., Zhang W., Zhang M. (2012). Asymmetric Influence of Egocentric Representation onto Allocentric Perception. J. Neurosci..

[40] Sosa G.W., Lagana L. (2019). The Effects of Video Game Training on the Cognitive Functioning of Older Adults: A Community-Based Randomized Controlled Trial. Arch. Gerontol. Geriatr..

[41] Park D.C., Reuter-Lorenz P. (2009). The Adaptive Brain: Aging and Neurocognitive Scaffolding. Annu. Rev. Psychol..

[42] Salthouse T.A. (2019). Trajectories of Normal Cognitive Aging. Psychol. Aging.

[43] Folstein M.F., Folstein S.E., McHugh P.R. (1975). “Mini-Mental State”: A practical method for grading the cognitive state of patients for the clinician. J. Psychiatr. Res..

[44] Cullen B., Fahy S., Cunningham C.J., Coen R.F., Bruce I., Greene E., Coakley D., Walsh J.B., Lawlor B.A. (2005). Screening for Dementia in an Irish Community Sample Using MMSE: A Comparison of Norm-adjusted versus Fixed Cut-points. Int. J. Geriatr. Psychiatry.

[45] Caissie A.F., Vigneau F., Bors D.A. (2009). What Does the Mental Rotation Test Measure? An Analysis of Item Difficulty and Item Characteristics. Open Psychol. J..

[46] Kozhevnikov M., Hegarty M. (2001). A Dissociation between Object Manipulation Spatial Ability and Spatial Orientation Ability. Mem. Cogn..

[47] Klatzky R.L., Freksa C., Habel C., Wender K.F. (1998). Allocentric and Egocentric Spatial Representations: Definitions, Distinctions, and Interconnections. Spatial Cognition.

[48] Billinghurst M., Weghorst S. (1995). The Use of Sketch Maps to Measure Cognitive Maps of Virtual Environments. Proceedings of the Virtual Reality Annual International Symposium ’95, Research Triangle Park, NC, USA, 11–15 March 1995.

[49] Meade M.E., Wammes J.D., Fernandes M.A. (2018). Drawing as an Encoding Tool: Memorial Benefits in Younger and Older Adults. Exp. Aging Res..

[50] Uttal D.H., Meadow N.G., Tipton E., Hand L.L., Alden A.R., Warren C., Newcombe N.S. (2013). The Malleability of Spatial Skills: A Meta-Analysis of Training Studies. Psychol. Bull..

[51] Garcia-Navarra S., Solares L., Mendez M. (2025). Virtual Reality and Mixed Reality in the Assessment of Spatial Memory. Explor. Digit. Health Technol..

